# A unique temporary collateral pathway between carotid-vertebrobasilar arteries in a carotid dissection patient

**DOI:** 10.1186/s12883-020-01651-1

**Published:** 2020-03-17

**Authors:** Xiaogang Liu, Bing Li, Ying Liu, Hongliang Wu, Huilong Zhang, Lianwei Dou, Chuanyu Liu

**Affiliations:** 1 Departments of Interventional radiology, The Affiliated Yantai Yuhuangding Hospital of Qingdao University, Yantai, Shandong 264000 People’s Republic of China; 2 Departments of Neurology, The Affiliated Yantai Yuhuangding Hospital of Qingdao University, Yantai, Shandong 264000 People’s Republic of China; 3 Departments of Rheumatology, The Affiliated Yantai Yuhuangding Hospital of Qingdao University, Yantai, Shandong 264000 People’s Republic of China

**Keywords:** Internal carotid artery dissection, Collateral circulations, 3-dimensional rotational angiography, Ischemic stroke

## Abstract

**Background:**

In adults, the anastomosis between carotid and vertebrobasilar arteries is usually the posterior communicating artery, sometimes the primitive trigeminal artery. In this case, the basilar artery fed the internal carotid artery through the pontine-to-tentorial artery anastomosis after severe stenosis from traumatic carotid dissection.

**Case presentation:**

A 32-year-old female was diagnosed with ischemic stroke caused by traumatic carotid artery dissection. Aspirin (100 mg/day) and clopidogrel (75 mg/day) were prescribed. Digital subtraction angiography performed 6 days after stroke onset showed a dissection in the cervical segment of left internal carotid artery with severe local stenosis, and a collateral pathway from BA to the cavernous segment of internal carotid artery through the lateral pontine and tentorial artery. Without interventional therapy, clinical symptoms improved significantly within 10 days after onset. At 3-month follow-up, left common carotid artery angiography showed the stenosis had been significantly improved with a residual aneurysm. There was no collateral pathway between carotid-vertebrobasilar arteries, and a residual small artery originated from the posterior vertical segment of cavernous internal carotid artery. The small artery was clearly visualized by 3-dimensional rotational angiography and identified the tentorial artery.

**Conclusion:**

To the author’s knowledge, this is the first report of a collateral pathway between carotid vertebrobasilar arteries through the pontine-to-tentorial artery anastomosis. Meanwhile, tentorial artery origination directly from the cavernous segment of internal carotid artery is rare and easily mistaken for persistent primitive trigeminal artery. 3-dimensional rotational angiography can provide sensitive and accurate diagnostic assessment of the small artery, and may be a useful tool for screening of abnormal small arteries.

## Background

In adults, the anastomosis between carotid and vertebrobasilar arteries is typically the posterior communicating artery. However, persistent primitive trigeminal artery (PTA), which provides blood supply to the hindbrain during embryonic development and is usually obliterated by 11.5- to 14-mm embryonic stage [1], is the most common of primitive anastomoses that may persist into adulthood [2]. When common carotid artery (CCA) occlusion occurs, the vertebral artery can supply blood to the external carotid artery (ECA) through its branches, and retrogradely to CCA and internal carotid artery (ICA). In this case, blood flow was significantly reduced after left ICA dissection, and the basilar artery supplied blood to cavernous and distal ICA through lateral pontine and tentorial arteries. To our knowledge, this rare pathway between carotid-vertebrobasilar arteries has not been reported. After improvement of ICA stenosis, arteries connecting the cavernous segment of ICA became very small. The pathway was clearly visualized by 3-dimensional rotational angiography (3DRA) and identified the tentorial artery.

The tentorial artery, also known as marginal tentorial artery, or medial tentorial artery, usually originates from meningohypophyseal trunk (MHT) and occasionally directly originates from the posterior curvature of cavernous segment of ICA. This artery is more significant than usual when it is involved in the blood supply of neoplasms or vascular malformations in the vicinity of tentorium cerebelli [3]. In this case, it becomes prominent because it is involved in the blood supply of the internal carotid artery.

## Case presentation

**Fig. 1:**
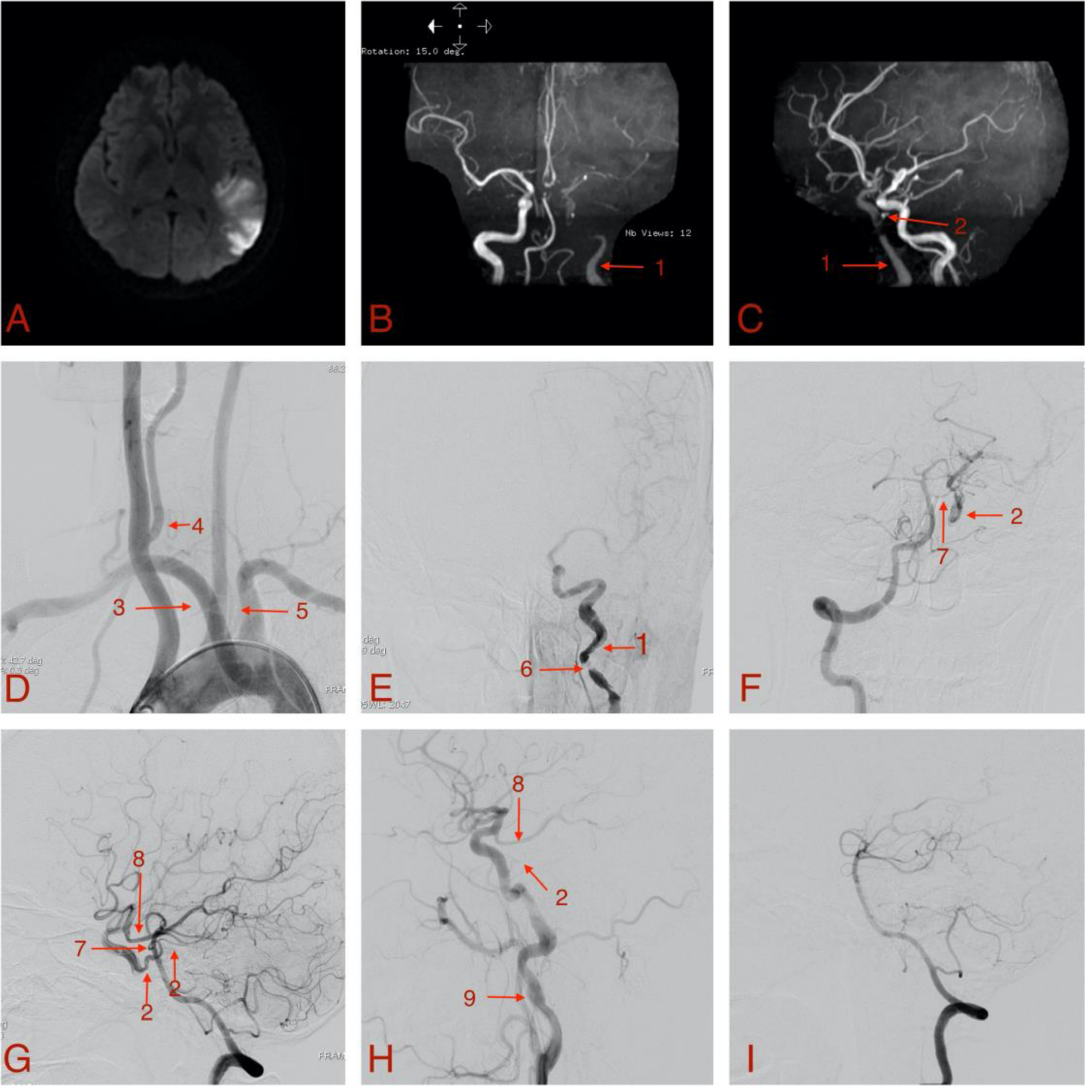
Diffusion Weighted Imaging (**a**) and magnetic resonance angiography (**b**, **c**) at 24 h after stroke onset, Digital subtraction angiography at 6 days after stroke onset (**d**, **e**, **f**, **g**) and at 3 months after stroke onset (**h**, **i**). **a** Diffusion Weighted Imaging showed moderate-sized infarcts involving the left frontal-parietal cortex; **b** Anteroposterior image of magnetic resonance angiography; **c** Oblique image of magnetic resonance angiography; **d** Aortic arch angiogram; **e** Anteroposterior view of left common carotid artery angiography; **f** Anteroposterior view of right vertebral artery angiography; **g** Lateral view of right vertebral artery angiography; **h** Lateral view of left carotid angiography; **i** Lateral view of right vertebral artery angiography. 1: the left internal carotid artery; 2: the tentorial artery; 3: the aberrant right subclavian artery; 4: the right vertebral artery originated from the right common carotid artery; 5: the left vertebral artery originated from aortic arch; 6: the formation of carotid dissection; 7: the lateral pontine artery; 8: the posterior communicating artery; 9: the internal carotid artery aneurysm

A 32-year-old female with no history of hypertension, diabetes, pregnancy induced hypertension, smoking, drinking or drug abuse, fell from bed 3-months postpartum and landed on her head, immediately experiencing global aphasia, dizziness, and paralysis of right limbs for 10 min. After 2 h, the patient continued experiencing anomic aphasia, repetition impediment, and dyslexia. No cerebral hemorrage was found by computed tomography (CT), and thrombolysis was not performed because of the recent head trauma. Due to the mild symptoms, aspirin (100 mg/day) and clopidogrel (75 mg/day) were given, and no emergency endovascular treatment was performed. Twenty-four hours later, Magnetic resonance imaging (MRI) showed moderate-sized infarcts involving the left frontal-parietal cortex (Fig. 1a), and magnetic resonance angiography (MRA) showed that there was no development from the petrous segment of the left ICA to the lacerum segment (Fig. 1b, c). The basilar artery was connected to the left cavernous ICA through the tentorial artery, and the distal part of the internal carotid artery is developed lightly.

Because pregnancy will lead to the occurrence or aggravation of connective tissue diseases and endocrine diseases, we have improved the relevant laboratory tests to exclude the vasculitis caused by such diseases, so that it is more likely to have dissection in trauma. Complete blood count, erythrocyte sedimentation rate (ESR), blood urea nitrogen (BUN), creatinine, glucose, total protein, albumin, thyroid function tests, antinuclear antibody (ANA), antiextractable nuclear antigen (ENA) antibodies, anti-double stranded DNA (dsDNA) antibodies, complement, immunoglobulins, and rheumatoid factor (RF) were normal.

Digital subtraction angiography (DSA) was performed 6 days after stroke onset. Arch aortography (Fig. 1d) showed aberrant right subclavian artery, right vertebral artery arising from right CCA, and left vertebral artery arising from the aortic arch. Left CCA angiography (Fig. 1e) showed dissection forming in the proximal initial segment of left ICA with severe local stenosis. Right vertebral artery angiography (Fig. 1f, g) showed a collateral pathway from the basilar artery to the cavernous sinus of ICA through the lateral pontine and tentorial arteries, and another collateral pathway from left posterior cerebral artery to distal ICA through the posterior communicating artery. Based on this information, medication was continued without interventional therapy, and, 10 days from onset, clinical symptoms improved significantly.

**Fig. 2:**
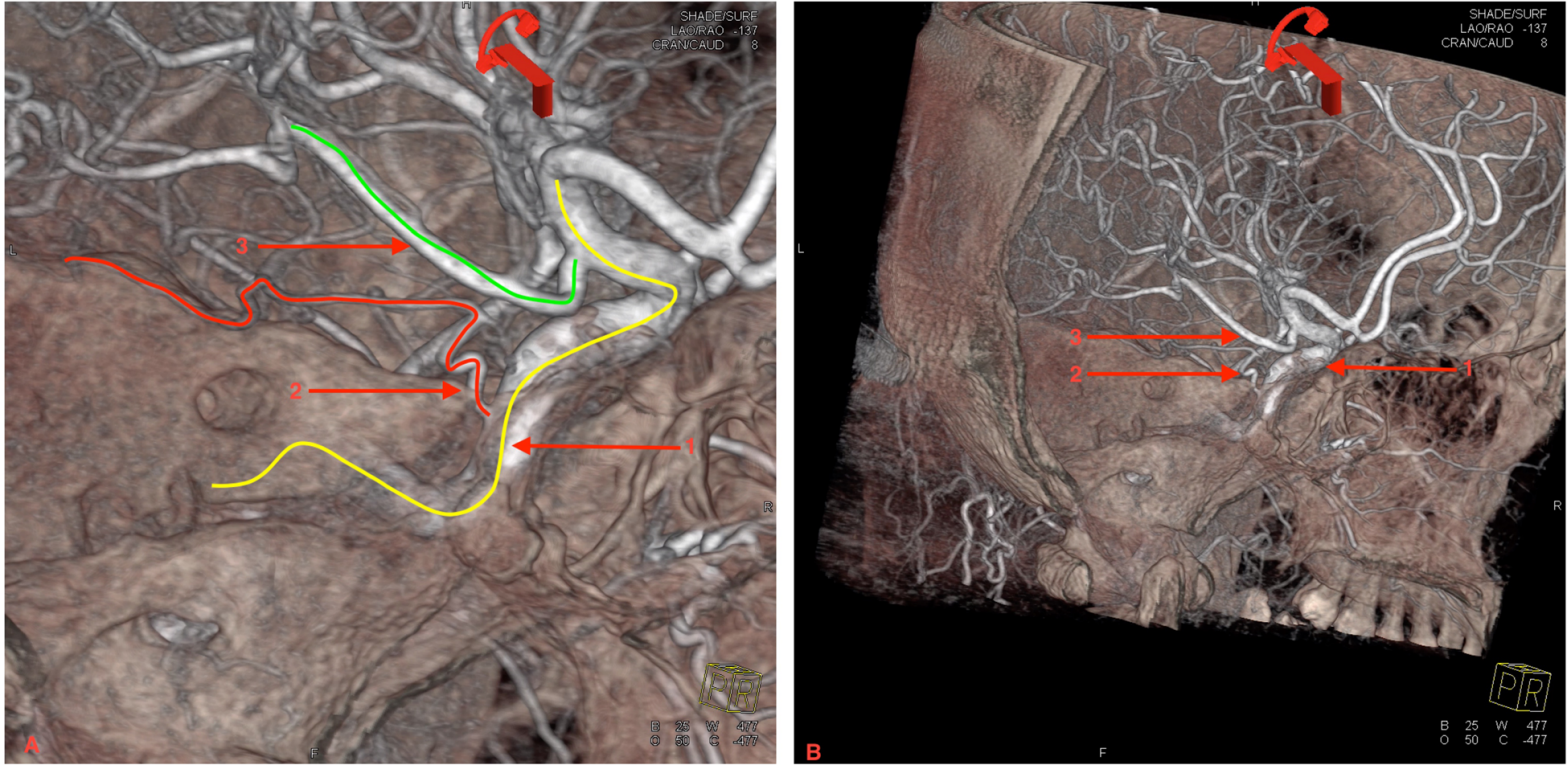
**a** Local enlargement of **b**) The 3-dimensional rotational angiography of left internal carotid artery 3 months after stroke onset. The post-processing image clearly showed the complete path of the tentorial artery. 1/Yellow line: left internal carotid artery, 2/Red line: the tentorial artery, 3/Green line: the left posterior communicating artery

Repeat DSA was performed at 3-month follow-up. Left CCA angiography (Fig. 1h) showed residual aneurysm in the area where carotid dissection occurred originally, with no significant stenosis. A small artery originated from the posterior vertical segment of cavernous ICA, and the distal segment was not clearly visible. Additionally, the posterior communicating artery originated from distal left ICA. Subsequently, 3DRA of left ICA (Fig. 2) was performed, and the tentorial artery course was clearly visualized, starting from the cavernous ICA and circumscribing retrogradely and superiorly, ending at the cerebral tentorium with several branches. No collateral pathway supplying blood to ICA was seen with right vertebral artery angiography (Fig. 1i). The patient remained clinically stable with no further treatment, and the patient was very satisfied with the treatment.

## Discussion and conclusions

Post-traumatic carotid dissection resulted in severe ICA stenosis and marked blood flow reduction, leading to aphasia and right limb paralysis. However, when the collateral pathway began supplying blood, symptoms improved. The anastomosis between the pontine and tentorial arteries became a major posterior-to-anterior blood supplier.

There is no substantial agreement about the medical management of carotid artery dissection. We did not confirm the diagnosis of carotid dissection until the DSA on the 6th day after onset. Since the patient’s symptoms gradually improved, we continued to use antiplatelet therapy.

In only 3% of the cases, the tentorial artery arises directly from cavernous ICA [4]. In this case, the tentorial artery arose directly from posterior vertical segments of cavernous ICA, which is quite rare. A previous study showed the majority of MHT branches (including tentorial, dorsal meningeal, and inferior hypophyseal arteries) anastomosed with opposite mates and dural branches of the ECA [5]. However, there are no reports of contralateral ICA or ipsilateral ECA supplying blood to the affected ICA through this anastomosis.

Tentorial arteries are usually small and difficult to detect using conventional angiography. Underlying pathological circumstances increasing blood flow (such as dural arteriovenous fistula, arteriovenous malformations, hemangioblastomas, and malignant gliomas) make the vessel more visible on angiography [6]. Sato et al. report a patient with symptomatic bilateral vertebral artery occlusion where MRA showed marginal tentorial arteries running along the tentorial edge and anastomosing with each posterior cerebral artery as collateral circulation, which was barely visible on CCA angiography [7]. The tentorial artery involved in collateral blood supply is quite rare and difficult to detect because of its small size. But in this case, the tentorial artery was quite significant with reverse flow, and involved in the collateral blood supply of the anterior circulation when the ICA flow decreased significantly. After the improvement of the ICA stenosis, this artery became small and the blood flow direction returned to normal.

The complete MRA examination 24 h after the stroke onset showed that the petrous segment of the internal carotid artery was occluded or severely narrowed, and the distal blood flow was supplied by the tentorial artery, which plays an important role in the early stage of the disease, so as to avoid high-risk endovascular treatment in the acute stage.

Similar to the persistent PTA, the tentorial artery arises from the cavernous ICA directly and without branches. PTA is the most common variant of carotid-vertebrobasilar anastomoses, with an estimated prevalence of 0.1–0.6% [8]. While classic persistent PTA extends from ICA to the basilar artery, PTA variant continues as a cerebellar artery without basilar connection [9]. However, in these cases, PTA variants are not connected to the vertebral or basilar arteries, and collateral blood supply cannot be offered from the vertebrobasilar artery system when ICA is stenosed or occluded. In an anatomical study, persistent PTA branched out to the pons on its way to the basilar artery rather than anastomose with lateral pontine arteries [10]. To our knowledge, there are no reports of PTA anastomosis with lateral pontine arteries. In this case, after receiving the lateral pontine artery blood supply, this vessel not only supplied blood to ICA reversely, but also developed anteriorly at the distal end of the anastomosis point. Reviewing the literature carefully, PTA arteries terminated in the basilar artery or other arteries supplying blood to the cerebellum but did not continue moving forward after anastomosing. Therefore, the artery involved in collateral blood supply in our case was not PTA.

After improving carotid stenosis, the tentorial artery arising from the posterior vertical segment of cavernous ICA became smaller and harder to identify with conventional angiography. 3DRA clearly shows the path of small blood vessels from many angles, and helped us accurately identify the tentorial artery by preserving the image of bone and dural structures. Therefore, 3DRA is helpful in showing small, tortuous vascular pathways and their relationship with peripheral blood vessels and other structures, thus providing a reliable basis for diagnosis and treatment.

Abnormal aortic arch is also very rare. Right subclavian artery originating distal to the left subclavian artery, in combination with the right vertebral artery arising from the right CCA, has only been documented in 10 cases [11]. When the origin of a single vertebral artery is abnormal, the incidence of other abnormal vertebral origins increases [12]. However, the abnormal aortic arch had no obvious clinical significance in the diagnosis or treatment of this case.

This is a rare case in which the basilar artery fed the ICA through the pontine-to-tentorial artery anastomosis after severe stenosis from traumatic dissection. Tentorial artery origination from the cavernous segment of ICA is rare and easily mistaken for PTA. 3DRA identified the origination point and traced the entire vascular pathway.

## Data Availability

All data generated or analyzed during this study are included in this published article.

## Abbreviations

3DRA: 3-dimensional rotational angiography
CCA: Common carotid artery
DSA: Digital subtraction angiography
ECA: External carotid artery
ICA: Internal carotid artery
MHT: Meningohypophyseal trunk
PTA: Primitive trigeminal artery
